# What Do Medical Students Learn about Community Care from Discussions with General Physicians? A Thematic Analysis

**DOI:** 10.3390/medicina59101855

**Published:** 2023-10-19

**Authors:** Ryuichi Ohta, Nozomi Nishikura, Chiaki Sano

**Affiliations:** 1 Community Care, Unnan City Hospital, 96-1 Iida, Daito-cho, Unnan 699-1221, Japan; nozonishi0702@gmail.com; 2 Department of Community Medicine Management, Faculty of Medicine, Shimane University, 89-1 Enya cho, Izumo 693-8501, Japan; sanochi@med.shimane-u.ac.jp

**Keywords:** community care, medical education, thematic analysis, rural medicine, student perceptions, experiential learning, constructivism, COVID-19 impact, dialogues with physicians, evolving healthcare

## Abstract

*Background and Objectives*: Sustainable healthcare is fundamentally rooted in community medicine education. The COVID-19 pandemic disrupted global advancement in integrating traditional teaching and experiential learning. Additionally, an alarming decline in interest in community care has been observed among senior medical students. Here, we examined the perspectives on community care obtained from conversations with general physicians in rural medical universities. *Materials and Methods*: Using a constructivist lens, a qualitative methodology was employed to examine the perceptions of second-year medical students from Shimane University Medical School regarding community care, informed by dialogues with general physicians. We conducted a thematic analysis at Shimane University, Japan, an area known for its aging population. In 2023, 116 second-year students participated, none of whom had prior formal training in community care. The study was structured into three phases: (1) Pre-education: Students wrote essays about their initial understanding of community care, its advantages, disadvantages, and potential improvements; (2) Dialogue: Grouped by topic, students engaged in discussions that culminated in a comprehensive session with general physicians; and (3) Reflection: After discussions, students wrote essays reflecting any shift in their views on community care. A thematic analysis of essays from the pre-education and reflection phases provided a comparative perspective on the students’ understanding. *Results*: Five dominant themes emerged from the thematic analysis: (1) Re-evaluating community care: Recognizing diversity and addressing societal challenges; (2) Interdisciplinary collaboration: Promoting shared roles and teamwork; (3) Learning and practice: Emphasizing hands-on experience and self-reflection; (4) Technological influence: The mutual relationship between community care and technological advancements; and (5) Challenges and resolutions: Identifying problems and crafting solutions. *Conclusions*: This study sheds light on the evolution of medical students’ views on community care and underscores the importance of continuous adaptation in medical education programs.

## 1. Introduction

The cornerstone of sustainable healthcare, particularly at the grassroots level, is education in community medicine. Understanding community care provides the foundation for robust health systems and serves as a beacon to inspire medical students [1]. A deeper understanding of community care can incentivize medical students to serve at the forefront of communities and foster a culture of community-centric care [2,3].

Medical education focusing on community care has undergone considerable advancements worldwide. Educational methodologies have been devised in multifaceted ways, integrating conventional classroom teaching with experiential learning in community medical institutions [4,5]. This holistic approach ensures students grasp theoretical concepts and understand their practical implications [6].

However, the recent COVID-19 pandemic posed unprecedented challenges to this learning mode [7]. With the looming threat of the spread of the virus, experiential learning in real community settings became substantially hampered, depriving students of invaluable on-ground experience [8,9].

Furthermore, an emerging trend has been observed in which, paradoxically, as medical students ascend the academic ladder in universities, their inclination towards community care diminishes [10]. Several factors contribute to this paradox, such as limited exposure to community care settings, reduced interactions with general physicians, and, at times, a misplaced perception stemming from the views of specialized medical professionals within university settings [11].

Emphasizing the essence of community care, medical students should be consistently engaged with general physicians [12]. Such interactions are pivotal for their practical education and to ensure that the spirit and sustainability of community care persist [13]. Considering the Volatility, Uncertainty, Complexity, and Ambiguity era, exploring diverse educational methodologies for community care and extending beyond the traditional boundaries of classroom and on-site learning are imperative [14].

One promising avenue is to foster dialogue and discussion on community care among medical students, their peers, and general physicians. This interactive approach deepens the understanding of community care and acts as a catalyst, triggering novel ideas and bolstering the learning curve [15]. Therefore, in this study, we aimed to investigate medical students’ learning outcomes concerning community care, mainly focusing on the insights gained through discussions and dialogues with general physicians in rural medical universities.

## 2. Materials and Methods

This qualitative study was comprised of second-year medical students at Shimane University Medical School and was conducted to clarify what medical students learned about community care through dialogue with general physicians at rural medical universities. This research stood on relativist ontology and constructivist epistemology to investigate their perceptions of and motivations for learning with respect to context. The purpose demands inductive approaches for detecting learning content in discussions and dialogue about community care. Therefore, we chose an inductive thematic analysis for this study.

### 2.1. Setting

This study was conducted among medical students at the Faculty of Medicine, Shimane University, Japan. Shimane is a rural area in southwestern Japan, and its population is one of the oldest age-wise among all prefectures, with 34.3% of the total population of 674,000 people aged 65 years and older [16].

### 2.2. Participants

In 2023, 116 second-year medical students were enrolled at Shimane University Medical School. They had never officially learned about community care in the university curriculum. All participants agreed to participate in this research, and their essays were used in the analysis of the present study [16].

### 2.3. Class of Community Care with Dialogue and Discussion among Peers and General Physicians

Educational discussions were conducted among participants and general physicians to educate the former. This educational session lasted 120 min and consisted of a pre-education stage, a discussion and dialogue stage, and a reflection stage. This session was in line with flipped classroom educational methods and theories of constructivism, collaboration, context, and self-directed learning [17,18]. 

In the pre-education stage, participants reflected on their learning of community care based on their prior knowledge and families’ experiences in community care. Based on these reflections, they wrote essays on the advantages and disadvantages of community care and revision points for better community care. The word limit for the essays was more than 200 and less than 400 words in Japanese. They submitted essays to general physicians 1 week before the beginning of the discussion stage.

In the discussion and dialogue stages, participants were divided into groups of five or six members. The discussion session encouraged dialogue on the merits and drawbacks of community care as well as proposing points for enhancing community care. Each topic was discussed in each group for 20 min based on their essays from the pre-education stage. After discussions among the participants, each group presented the results of their discussions and discussed them with general physicians. After discussions on all three topics, general physicians summarized the discussions and present conditions of community care in Shimane, Japan.

In the reflection stage, participants reflected on their learning about community care based on the discussion and dialogue stages. Based on this reflection, they wrote essays on the changes in their perception of community care. The word limit of the essays was more than 200 and less than 400 words in Japanese. They submitted essays to general physicians 1 week after completing the discussion stage.

### 2.4. Data Collection

#### 2.4.1. Essay Regarding Community Care

The pre-education and reflection essays were analyzed to clarify the research purpose. The pre-education essays consisted of three sections on the advantages and disadvantages of community care and revision points for better community care. The reflection essays comprised one section on changes in the perception of community care. The pre-education essays revealed the participants’ recognition of community care without discussion interventions. The reflection-stage essays described changes in the participants’ perceptions of community care through discussion interventions. The essay contents were submitted to researchers in the form of Excel sheets.

#### 2.4.2. Data Analysis

Thematic analysis, as framed by Braun and Clarke (2006), was employed to examine the essays, offering a structured methodology to identify and analyze patterns within the data. This qualitative approach was deemed most suitable for the present study, given its capacity to deeply explore and understand the students’ evolving perceptions of community care through their reflective essays and group discussions. Furthermore, this methodology facilitates the scrutiny of subjective experiences and perceptions, allowing for detailed insights into how dialogues with general physicians in a rural setting impacted medical students’ understanding and considerations about community care [19].

In the initial stage of familiarization with the data, two researchers (RO and NN) read the essay transcripts multiple times, noting initial ideas. In generating the initial codes, RO and NN conducted systematic coding across the entire data set, generating relevant codes for the data pertaining to the research question. Through discussions between RO and NN, codes were sorted into potential themes, and all relevant coded data extracts were collated within the identified themes. The themes were then reviewed for the coded extracts and the entire data set. In case of any conflicts with the researcher, themes were refined, split, combined, or discarded. Each theme was refined in terms of scope and focus. Clear definitions and names were developed for each theme. At this stage, RO, NN, and CS discussed the content until a consensus was reached. 

Regarding our sample size, while 116 might seem significant for a qualitative study, it was chosen to encompass the entirety of the second-year cohort’s experiences based on previous research, showing enough sample size of thematic analysis for document analysis [20]. This comprehensive approach aimed to provide a more in-depth and nuanced data set, given the unique nature of our cohort.

Regarding data saturation, RO and NN meticulously tracked the emergence of new themes as we analyzed the essays. By the time RO and NN had analyzed essays from 80 students, no new themes were surfacing, indicating data saturation. To illustrate, initial essays introduced approximately 10 unique themes, with subsequent essays adding fewer themes. By the 80th essay, the addition of new themes had plateaued. While RO and NN acknowledged saturation at this point, analyzing all 116 essays ensured RO and NN had not overlooked any nuanced or less common perspectives. RO wrote the findings with vivid and compelling examples to produce the report. The relationship between the analysis, research question, and literature was emphasized. Data were managed using NVivo 11.

#### 2.4.3. Reflexivity

This study was conducted collaboratively through interactions between researchers and participants. The research team had diverse expertise and perspectives on rural community care. RO is a family physician and public health professional with a master’s degree in public health and family medicine and has experience researching rural community healthcare and medical education qualitatively and quantitatively. NN is a family physician and public health professional with a master’s degree in medical education and has experience researching rural community healthcare and medical education qualitatively and quantitatively. CS is a medical educator and professor at a medical university who graduated from a medical university specializing in community healthcare management and education. To minimize bias, each idea related to community care was discussed by analyzing the research content of the individual data. Alternative viewpoints were explored in the data interpretation stage.

#### 2.4.4. Ethical Consideration

The Unnan City Hospital Clinical Ethics Committee approved the study protocol (No. 20230009).

## 3. Results

### 3.1. Results of the Thematic Analysis

Through a comprehensive thematic analysis of discussions concerning community care, five key themes emerged that illuminated medical students’ learning experiences. These themes provide insights into the evolving perspectives on community care and highlight their significance in modern medical education. The five themes reassess the essence and role of community care, deepening interdisciplinary collaboration in medical teams, learning and practice in community care, the future of technology and community care, and concrete challenges and solutions in community care (Table 1).

### 3.2. Reassessing the Essence and Role of Community Care

#### 3.2.1. Diversity in Community Care

By learning about community care through educational interventions, the medical students acknowledged the multifaceted nature of community care, underscoring its various forms and manifestations. The medical students previously considered community care to be restricted to rural areas and unrelated to urban areas. Learning about community care broadened their understanding of community care. One participant stated, “It was good to discuss community healthcare from various perspectives. The image of community healthcare had been one of rural or mountainous areas, but I have broadened my perspective to realize that community healthcare also exists in urban areas. Some areas provide access to clinics, even within mountainous regions. In contrast, others, where buses do not operate, have been unable to provide access to clinics or other healthcare facilities” (Participant 6). 

Considering community care, the participants realized that differences in social resources in rural and urban areas could affect healthcare quality and lead to different problems. The participants realized that each region required a different approach for improvement. One of the participants stated, “Rather than focusing solely on healthcare in rural and remote locations, I felt it was important to understand that community healthcare exists in each region. As cultures and problems vary by region, it is necessary to consider each area individually. Regarding the noted merit of community healthcare of being close and intimate with the residents, I felt that I wanted to value this, no matter where I worked” (Participant 22).

#### 3.2.2. Contributions to Social Challenges in Community Care

The medical students reflected on the pivotal role that community care plays in addressing social challenges and providing comprehensive patient-centered care. They understood various issues related to aging societies in providing comprehensive care. Aging populations and congestion conditions affect community care in urban areas. However, rural community care has issues such as lack of healthcare professionals and isolated older people. One participant stated, “Community healthcare requires the provision of comprehensive medical care based on the characteristics and needs of the community. Factors such as the population composition of the area, geographical conditions, and cultural backgrounds influence healthcare provision. Community health care must consider these factors and provide appropriate and continuous medical care to people in the area. Moreover, community healthcare emphasizes the treatment of diseases and prevention and health promotion. Understanding the health status of the people in the area and focusing on disease prevention, early detection, and improving lifestyle habits are vital. Community healthcare requires a holistic approach that considers individual patients and the overall health of the entire community” (Participant 26).

To improve community care, medical students should learn patient-centered care regarding culture, indigenous lives, and relationships with others in their communities. The participants realized that patient care quality was assessed using various frameworks, such as readmission rates to hospitals in comprehensive care by general physicians. They learned that the quality of patient care should be assessed from various perspectives. One of the participants stated, “The fact that general physicians could provide evidence-based data, such as the reduction in readmission rates, in response to the question of whether they genuinely contribute to community healthcare was highly meaningful for me, who wants to contribute to society as a doctor in community healthcare in the future. Until now, the concept of community healthcare has had a vague image for me, but I feel that I have come to grasp it with a slightly clearer picture” (Participant 45).

### 3.3. Deepening Interdisciplinary Collaboration in Medical Teams

#### 3.3.1. Forming Collaboration and Role Sharing

The importance of teamwork and shared responsibilities was highlighted, emphasizing the value of each team member’s contributions. The medical students realized that effective communication with citizens is essential for effective community care. They realized that to work in communities as medical professionals, they needed to learn how to communicate with citizens and patients from a biopsychosocial perspective. One participant stated, “In today’s discussion, where diverse opinions were presented, I understood that there is no comprehensive definition of community healthcare. Rather, community healthcare refers to a system that provides medical care according to the individual needs of each region” (Participant 34). Another participant stated, “Communication skills are essential for community healthcare, so I want to strive to think from the patient’s perspective and communicate in that way at all times” (Participant 37).

Interprofessional collaboration is another topic of communication. Through discussions, the medical students learned that effective community care cannot be accomplished only by physicians. They realized that better physicians hope to learn more about inter-professional collaboration and how to communicate with other healthcare professionals from different perspectives. One participant stated, “I realized the importance of coordinating with surrounding clinics and hospitals beyond my own to deliver medical care to the entire community. Therefore, I feel that it is necessary to maintain good relationships with patients and foster communication with all the medical staff in the area. Referral letters were also mentioned, and I thought that writing them in a way the recipient can understand might also be a communication skill” (Participant 29).

#### 3.3.2. Effects and Challenges of Collaboration

While the benefits of interdisciplinary collaboration were apparent, students identified several challenges, suggesting the need for refined collaborative strategies. The medical students realized effective collaboration with multiple professionals was essential for patient and community care. Their understanding of community care expanded from that of a single person to the quality of community care. One participant stated, “In learning about community healthcare going forward, what I want to prioritize is collaboration and communication with the community. Building close relationships with the people in the community is essential for community healthcare. By listening to the voices of the people in the community and reflecting on their needs and opinions, I believe that more effective medical services can be provided. Moreover, collaboration with professionals is vital for community healthcare. Doctors, nurses, public health workers, social welfare workers, and other professionals must collaborate to build a system that responds to the community’s medical needs. Cooperation among people with different levels of expertise is required to support the overall health of the community” (Participant 26).

However, the participants realized that various limitations to effective collaboration existed. In rural contexts, the lack of healthcare resources and aging physicians are considered critical. In urban areas, the gaps and differences between medical institutions are considered limitations to interprofessional collaboration. One participant stated, “My hometown is not particularly rural, but there are only two internal medicine clinics in the town. Therefore, if one needs to see an ophthalmologist or orthopedic surgeon, one must travel to the neighboring town by train or car. Additionally, some private practitioners are aging, and I wonder how long they will continue to practice. While one can get to the hospital by car or train, I thought it might be especially burdensome for the elderly, and it may not be easy for them to go casually and have something checked. It might be unavoidable that there are differences in medical facilities between regions, but I thought it would be good if there were initiatives, such as running regular buses, to ensure that everyone could receive appropriate medical care” (Participant 49).

### 3.4. Learning and Practice in Community Care

#### 3.4.1. Importance of Practical Learning and Reflection

Practical experience in community care settings is crucial as it provides real-world insights and promotes hands-on learning. The medical students had the opportunity to reflect on their community care experiences in their present and previous living conditions through family experiences. Through reflections, they realized that they could objectivize their experiences compared with other participants’ experiences and that community care had various forms in different contexts through concrete experiences. One of the participants stated “Through this lecture, discussion, and my own experience, I have come to feel that the meaning of community healthcare is not something rigidly defined in textbooks but can change according to the needs of the individual and the community. Therefore, when dealing with the theme of community healthcare, I believe it is necessary to discern what characteristics the community has, what needs exist, etc., and adopt a flexible attitude in learning about community healthcare” (Participant 41).

Furthermore, medical students could deepen their learning through discussions with colleagues and general physicians. The participants realized that each colleague had different ideas about community care based on different experiences of community care, and through discussion and sharing of such experiences, they could deepen their understanding of community care. One participant stated, “Regarding community healthcare, it was good to hear diverse opinions from others through group learning. When I heard about experiences in community healthcare, I realized that there was a wider variety of cases than I had imagined. There are many drawbacks, advantages, and areas for improvement in community health care. What stood out to me was a story about an experience with community healthcare, in which a presenter from another group talked about how good it was for a person who wanted to spend their last moments at home to receive home-based medical care as they wished. However, someone in my group who shared their experience with home-based medical care said that they did not get enough explanation about it, and did not understand it well, so they ended up choosing palliative care in the hospital” (Participant 46).

#### 3.4.2. Medical Students’ Career Formation in Community Care

The idea of engaging in community care significantly influenced the students’ career trajectories, with many considering it a potential specialty or focus. Through this course, the medical students became interested in community care and learned that community care can deal with various biopsychosocial issues. They realized that working in community care could be an attractive choice for professionals. One of the participants stated, “I was not aware that the role of general practitioners was interesting, so I am glad to have learned that. Going forward, while valuing my intentions, I want to become a doctor who can provide community care that considers the surrounding community residents” (Participant 20).

Simultaneously, the course clarified the limitations of working in community care. The medical students insisted that for effective learning about community care and working in such settings, medical systems for effective working for medical professionals should be secured. One participant stated, “There are current issues such as the uneven distribution of doctors and medical specialties, increased burden on physicians, and financial difficulties in medical institutions. It is also important to consider the serious impact of the aging population” (Participant 19). Another participant stated, “The uneven distribution of doctors is a problem, and it is important to find a way to resolve this. However, I think that it would be difficult to immediately dispatch doctors currently working in places where there is a shortage of physicians” (Participant 31).

### 3.5. Future of Technology and Community Care

#### 3.5.1. Utilization of Information Technology

The potential of information technology in enhancing community care practices was highlighted, from improving patient records to facilitating remote consultations. The medical students learned that medicine is divided because of the complexity of medical care and the aging society. They realized that the fragmentation of care, especially for older people, causes various problems in community care. They realized that various community healthcare professionals promote and facilitate information technology usage. One participant stated, “I thought it was good that there have been changes such as the advancement of online services, the improvement of regional inclusiveness, and the increase in patients’ ability to manage themselves, which have helped the field of regional healthcare suffering from staff shortages” (Participant 13).

Furthermore, they learned that information technology was implemented and driven by community care during the COVID-19 pandemic. They believed that information technology usage in community care could mitigate the fragmentation of care and differences in the levels of medical care depending on the place of residence. The participants insisted that information technology should be validated and used proactively in community care to improve the quality of care. One participant stated, “I thought that to enable people in mountainous regions to have equal access to medical resources, we must also create systems such as online medical consultations” (Participant 49).

#### 3.5.2. Importance of Collaborating with Technology

Medical students have learned that integrating technology with traditional medical practices is vital for ensuring efficient and up-to-date care. In this course, participants discussed how to use technology in community care. Advanced artificial intelligence (AI) has forced medical students to learn and use it more in their learning. One participant stated, “As AI and similar technologies continue to advance, I feel there is a growing need to study them. As a medical student involved in community healthcare, I have come to realize the importance of this knowledge. There are people around me who are well-versed in this area, while others have no clues. I believe that this might be an emerging field in the future” (Participant 26).

Responding to the need for AI, medical students realized they needed to learn more about AI in medical education. Additionally, using AI in students’ learning is believed to change their learning processes and enable more advanced learning. They insisted that AI learning should be applied more effectively in medical education. One participant stated, “I believe universities should increasingly create opportunities for students to learn about AI. As AI continues to evolve rapidly, medical education also needs to keep pace or might be left behind. Integrating AI into medical education appears to be a good move” (Participant 93).

#### 3.5.3. Evidence Development

The future of community care holds the potential for further research and evidence generation using technology, thus driving community care forward. The medical students realized that community care is complex and difficult to improve in terms of quality based on research, but various studies have clarified the quality of community care. They insisted that community care evidence should be driven more similarly to other medical fields and that such results could motivate more medical students to become general physicians in community care, leading to some solutions in community care. One participant stated, “In Shimane Prefecture, there is an observed imbalance in medical departments due to a shortage of doctors. I had thought that medical services for such patients might not be adequately provided. However, based on actual data, I have reconsidered that it would be more efficient to enhance general medical practice further, protect the health of local residents daily, and support a lifestyle that makes them less likely to become ill” (Participant 31). Another participant stated, “The question of whether general practitioners contribute to local medical care was also answered by providing data with solid evidence, such as a decrease in readmission rates. This information is precious for someone like me, who wants to contribute to society as a doctor who can aid in local healthcare in the future” (Participant 44).

### 3.6. Concrete Challenges and Solutions in Community Care

#### 3.6.1. Current Challenges in Community Care

The community care landscape presents various challenges, from administrative hurdles to resource limitations. The medical students learned to respect the backgrounds of each community. They realized that community backgrounds affect people’s healthcare and behavior, which could affect their health outcomes. They insisted that medical professionals should understand each community’s cultural and social aspects and engage in community care. One of the participants stated, “Regional healthcare takes into account the cultural background of the area, so there are aspects of it that one might not understand unless they visit the region. Therefore, to learn about regional healthcare, it is essential to visit the area, observe the local lifestyle, and witness the relationship between patients and doctors through hospital visits” (Participant 12).

For effective community care, the medical students learned about the effective usage of indigenous resources in each community. Cultural and social differences may create specific resources for community care. The medical students realized that medical professionals should detect specific resources in community care through collaboration with citizens and utilize them for better community care. One participant stated, “In the future, as I continue to study regional healthcare, I am aware that there are various forms of regional healthcare. I want to understand why specific healthcare practices have been established in a particular region. I plan to do so by focusing on the unique characteristics and resources of this area. While considering what "community" and "health" truly mean, I believe it is crucial to observe and feel these practices personally. I am committed to learning in this manner” (Participant 14).

#### 3.6.2. Proposals and Solutions

The medical students provided constructive solutions and strategies, pointing towards an optimistic future for community care. Through discussions in this course, medical students became more open-minded about community care. Initially, they emphasized the negative aspects of community care, such as the lack of a workforce and the poor environment of healthcare professionals. Through the discussion, they realized that many solutions could be proffered to revise the quality of community care as medical professionals. They acquired the attitude of considering a concrete method for revising community care. One participant stated, “As I delve deeper into studying regional healthcare in the future, what I consider crucial is not just memorizing superficial knowledge. Instead, I want to understand both sides: What are the advantages of certain aspects, and what are the underlying disadvantages? By contemplating how to improve these aspects, I aim to form my own opinions and beliefs. I believe that this approach to learning will lead to a better understanding” (Participant 18).

Furthermore, medical students were motivated to provide community care with respect to cultural and social aspects. Additionally, they realized that healthcare professionals should understand the needs of citizens in communities and match medical care to their needs for better and sustainable community care. One of the participants stated, “Previously, I believed there was a single correct answer to understanding regional healthcare. However, through this lecture, I realized that regional healthcare varies by region based on its specific needs. Therefore, it is essential to address these needs effectively. I also recognized the importance of collaboration among healthcare professionals, local residents, and multidisciplinary teams. I hope to provide medical care while paying close attention to the broad range of societal backgrounds” (Participant 25).

## 4. Discussion

In analyzing the experiences of medical students learning through dialogic engagement with community care, the identified themes elucidate the nuances and evolving dimensions of this area. Juxtaposed with previous literature, the importance of community care in contemporary medical education has become even more precise.

The shift in perception toward appreciating the diversity inherent in community care reflects a broader understanding of healthcare as both clinical and sociocultural. Recognizing the contributions of community care to social challenges indicates that it is perceived as a service and a dynamic interface between medical practice and societal needs [21]. Previous literature has often compartmentalized community care into distinct categories without accounting for its intrinsic diversity [22]. Our findings indicate a shift towards acknowledging the heterogeneity of community care, suggesting a broader, more holistic understanding of healthcare that transcends clinical perspectives. Acknowledging its role in addressing social challenges based on previous articles, the emphasis on diversity is novel, illuminating the evolving nuances of understanding community care [23]. However, this broad understanding may sometimes risk generalization, overlooking specialized community care nuances. The concept of community care has undergone considerable transformations over the years [24]. Our findings underscore the pivotal shift from compartmentalized perspectives toward appreciating the diverse fabric of community care. This changing perception recognizes the convergence of clinical and sociocultural facets of healthcare, marking a departure from traditional narratives [25]. Therefore, a holistic perspective should be juxtaposed with the intricate nuances of specialized areas to offer a comprehensive view of community care [26]. While our study builds on previous research, it also identifies gaps and avenues for future exploration, emphasizing the continuous evolution and depth of understanding necessary for community care.

The shift from isolated medical practices to collaborative approaches has been a topic of interest for several years. The hierarchical structure, which often places physicians at the apex with other healthcare professionals playing supportive roles, has been under scrutiny, especially given the rapidly evolving nature of healthcare needs [27]. Previous studies have emphasized the significance of interdisciplinary collaboration in healthcare [28,29]. Their research highlighted that the need for collaboration among different professionals intensifies as medical challenges become more complex, often cutting across various disciplines [28,29]. This argument is especially strong in community health, where challenges can be medical and sociocultural, necessitating the combined expertise of clinicians, social workers, and even community leaders [30]. Our study builds on this by highlighting collaboration and active “role-sharing.” Role-sharing moves beyond the simple notion of working together and suggests an environment in which roles are interchangeable based on the demand of the situation rather than strictly delineating professional boundaries [29]. However, as our study suggests, the fluidity of roles, while promising, comes with challenges. Blurring boundaries and responsibilities can lead to confusion, possible overlaps or gaps in care, and legal or ethical concerns [30]. While the shift towards a more collaborative and role-sharing model in community health is promising, it requires rigorous exploration, clear guidelines, and continuous training to ensure it is executed effectively and ethically.

The intricate relationship between community care, medical students’ learning, and aspirations offers a fresh perspective on how learning environments can influence future professionals. Tangible interactions, decision-making in real-life scenarios, and immediate reflections provide a robust platform for learning that often surpasses traditional classroom settings [31]. Our findings indicate that community care environments, with their diverse challenges and interactions, amplify experiential learning. They offer students a hands-on approach to medical practice and expose them to the broader sociocultural determinants of health, offering a holistic view of healthcare. As our study indicates, the ripple effect of these experiences extends to shape students’ career aspirations. The dynamics of community care, the immediate impact of interventions, and close interactions with patients and communities might sway students towards primary care, public health, or even health policy roles. However, while experiential learning equips students with practical skills, a robust theoretical framework is essential for critical thinking, innovation, and a deeper understanding of complex medical phenomena [32]. Community care undoubtedly plays a monumental role in shaping medical students’ learning and aspirations; however, it should be recognized as part of a broader educational ecosystem.

In the annals of medical history, a few things have transformed healthcare as profoundly as technology. Its ubiquity and permeation into various healthcare facets, especially community care, suggest a transformative shift in how care is perceived, delivered, and evaluated. Previous studies have demonstrated that technology is not merely a tool but also an integral component in enhancing healthcare outcomes, efficiency, and accessibility [33]. Rather than perceiving technology as a tool or an addition to healthcare, we are witnessing an era in which technology collaborates with medical practitioners [34]. This paradigm shift has profound implications. The focus on evidence development using technology suggests a move towards data-driven, research-based community care, ensuring that interventions are well-intended and effective [35]. Digital tools can aid in collecting, analyzing, and leveraging patient data to offer personalized care and optimize outcomes. However, overreliance on technology can risk turning care into a mechanical process devoid of nuances and personal touch, which often defines successful community care [36]. While technology undeniably promises to redefine community care, navigating this terrain with caution and awareness is imperative. Ensuring that technology is an ally rather than an overshadowing entity is crucial for preserving the holistic, patient-centric essence of community care.

The dynamic nature of community care presents a multitude of emerging and long-standing challenges. Our study emphasizes the proactive role of current medical students in navigating these issues, suggesting that they could be pivotal in reshaping the future of community care. While earlier research posited that identifying challenges is a preliminary step, it also emphasized the necessity of a transition from problem recognition to actionable solution development [37]. Our results concur with this viewpoint but introduce a fresh perspective: the increasing significance of medical students as agents of change. The innovative approaches proposed by these students offer a promising vision for community care. However, the journey from conceptualizing solutions to their practical application is often difficult [38]. For community care to truly evolve, an approach that combines problem-solving foresight with meticulous execution is imperative.

This study had several limitations. The participants were primarily recruited from a medical university. However, this approach may not capture the full spectrum of perspectives from diverse geographical regions or institutions using different pedagogical approaches. Given the qualitative approach used in this study, the derived insights and themes were largely interpretative. Therefore, these findings may not be generalizable to a broader population of medical students and other healthcare professionals. As with many qualitative research methodologies, an inherent risk of confirmation bias exists in which researchers may unintentionally prioritize data that align with their preconceived notions or hypotheses. Additionally, this study was conducted over a limited timeframe, which may not capture evolving perspectives or changes in community care paradigms over extended periods. Future studies should consider diversifying the participant sources, integrating mixed methods to combine qualitative depth with quantitative validation, and ensuring iterative data analysis to mitigate potential biases.

## 5. Conclusions

This study offers a profound understanding of medical students’ perceptions and learning experiences in community care. The emergent themes highlighted the evolving nature of community care and emphasized its significance in current medical education. As the field of medicine becomes more interdisciplinary, collaborative, and technologically integrated, community care stands out as a crucible for innovative practice and learning. These findings encourage medical educators to further integrate community care themes into curricula and foster environments that encourage reflective practice, technological adaptability, and problem-solving. This study offers a rich tapestry of insights into medical students’ perceptions and learning experiences in community care. The intricate interplay of evolving ideas becomes evident by juxtaposing these findings with existing literature. As medical education grapples with changing societal needs, technologies, and collaborative paradigms, community care has emerged as an invaluable focal point. Our findings validate and challenge existing pedagogical practices, underscoring the need for continuous evolution in medical curricula.

## Figures and Tables

**Table 1:** Results of the thematic analysis.

Theme	Concept
Reassessing the Essence and Role of Community Care	Diversity in Community Care
	Contributions to Social Challenges in Community Care
Deepening Interdisciplinary Collaboration in Medical Teams	Forming Collaboration and Role Sharing
	Effects and Challenges of Collaboration
Learning and Practice in Community Care	Importance of Practical Learning and Reflection
	Medical Students’ Career Formation in Community Care
Future of Technology and Community Care	Utilization of Information Technology
	Importance of Collaborating with Technology
	Evidence development
Concrete Challenges and Solutions in Community Care	Current Challenges in Community Care
	Proposals and Solutions

## Data Availability

The data sets used and/or analyzed in the current study may be obtained from the corresponding author upon reasonable request.
